# Face Pose Alignment with Event Cameras

**DOI:** 10.3390/s20247079

**Published:** 2020-12-10

**Authors:** Arman Savran, Chiara Bartolozzi

**Affiliations:** 1 Department of Computer Engineering, Yasar University, 35100 Izmir, Turkey; 2 Event-Driven Perception for Robotics, Istituto Italiano di Tecnologia, 16163 Genova, Italy; chiara.bartolozzi@iit.it

**Keywords:** event camera, dynamic vision sensor, low power, event-driven, face dataset, motion detection, face alignment, pose estimation, cascaded regression, extremely randomized trees

## Abstract

Event camera (EC) emerges as a bio-inspired sensor which can be an alternative or complementary vision modality with the benefits of energy efficiency, high dynamic range, and high temporal resolution coupled with activity dependent sparse sensing. In this study we investigate with ECs the problem of face pose alignment, which is an essential pre-processing stage for facial processing pipelines. EC-based alignment can unlock all these benefits in facial applications, especially where motion and dynamics carry the most relevant information due to the temporal change event sensing. We specifically aim at efficient processing by developing a coarse alignment method to handle large pose variations in facial applications. For this purpose, we have prepared by multiple human annotations a dataset of extreme head rotations with varying motion intensity. We propose a motion detection based alignment approach in order to generate activity dependent pose-events that prevents unnecessary computations in the absence of pose change. The alignment is realized by cascaded regression of extremely randomized trees. Since EC sensors perform temporal differentiation, we characterize the performance of the alignment in terms of different levels of head movement speeds and face localization uncertainty ranges as well as face resolution and predictor complexity. Our method obtained 2.7% alignment failure on average, whereas annotator disagreement was 1%. The promising coarse alignment performance on EC sensor data together with a comprehensive analysis demonstrate the potential of ECs in facial applications.

## 1. Introduction

Event cameras (ECs) are an emerging type of visual sensor that captures contrast changes at individual pixels. These changes are quantized into a stream of pixel-events each of which carries the time, coordinates and sign of the change. This approach leads to unprecedented capabilities as very high temporal resolution (in the order of μs), low latency, low power consumption (in the order of tens of mW [1]), and very high dynamic range (in the order of 140 dB [2]). ECs capture high frequency information free of motion blur in scenes with very bright, very dark, or uneven, illumination and can work on a low power budget, both at the sensor level, both thanks to the extremely compressed data representation, which allows for light weight computation, especially in scenarios with relatively low motion. These characteristics make ECs suitable for embedded applications in unconstrained environments, and where the information is mostly in the dynamic content of the scene, such as automotive, autonomous robotics and mobile. In this work we focus on coarse, but efficient, estimation of head pose, which can be used as pre-processing step for many face-related application, such as tracking, recognition and visually aided speech applications, needed for smooth human-device interaction.

The last decade witnessed a great progress in the face alignment field, thanks to the creation of comprehensive databases and advances in machine learning [3,4,5], however, to the author’s knowledge, this is the first time an EC is used for this task, also for the lack of adequate EC datasets. To fill this gap, we recorded an EC dataset with human subjects, characterized by large head rotations, varying movement speeds and repetitions, speaking intervals, and multi-human annotations, available upon request, and developed a coarse and efficient face pose alignment technique. A wide range of facial applications can benefit from our method even though we do not align very precisely for fine facial details. Existing EC-based voice activity detection [6], visual speech [7] or face recognition [8] can be implemented in a robust way against large head pose variations. However, our method does not aim at applications where fine detail alignment is necessary, as in 3D face reconstruction, facial performance capture or face transfer, unless it is used as a bootstrap initialisation technique in hybrid EC/frames systems.

The proposed method aims at a coarse but extremely efficient face alignment. At first, the detection of pose change activates the alignment, preventing unnecessary processing when the head doesn’t move. Alignment is then performed by regression cascade of tree ensembles, exploiting their superior computational efficiency [9,10] with respect to (possibly more accurate) state-of-the-art alignment methods based on deep neural networks (DNNs) [5,11,12,13,14,15,16,17,18,19,20,21,22,23,24,25,26,27,28,29,30,31]. In a scenario where energy efficiency is a matter of the utmost importance, a DNN-based alignment pre-processor might eclipse the energy efficiency advantage of ECs. Tree ensembles have also been suggested in a very recent study [32] instead of DNNs for the similar efficiency reasons, though for a different vision problem. Although DNNs have also been applied with ECs for different vision problems [33,34,35], those studies aim at benefits of ECs other than the energy efficiency, thus are computationally very demanding. The comparison of our method to human performance in facial landmark placement on the same dataset shows that extremely randomized trees (ERT) cascade applied directly on pixel-events—i.e., without facial image reconstruction and training on image datasets—has good accuracy. The method proposed in this manuscript implements a good trade-off between power efficiency and accuracy for different levels of head movement speeds, alignment with different initial localization uncertainty ranges, different resolution and different complexity levels of the predictor.

## 2. Related Work

### 2.1. Face Alignment

Face alignment, or facial landmark detection, is usually performed after face detection. It was studied for robust detection of key facial points and high precision alignment under highly varying conditions over many frame-based databases. These methods can be grouped into holistic, constrained local and regression-based methods [3,4]. Holistic methods explicitly model the whole face appearance and landmark-based face shape/pose patterns. Constrained local methods, instead, explicitly model local appearance patterns around landmarks. In both categories alignment can be realized by model fitting, or by regression-methods, that learn to predict landmark coordinates by implicitly imposing shape/pose constraints. Although considerable progress has been made with the two direct model fitting approaches, in general, regression methods offer better alignment and computation efficiency; more specifically, when realized in a cascade [3,4].

Cascaded regression is a gradient boosting machine [36], however, for improved alignment, pose/shape invariance is integrated by re-generating feature coordinates in accordance with the pose/shape prediction at the current stage. Dollár et al. [37] used cascaded random ferns to predict 2D affine pose transformations over three landmarks on the eye and mouth centers. Later their method was extended using more landmarks [38] to cope with occlusions. Non-linear least-squares fitting can be realized also as cascaded regression, leading to the supervised descent method for alignment [39]. Cao et al. [40] proposed shape regression with two-levels gradient boosting by efficient computation of shape-indexed feature coordinates. Extremely fast cascaded regressions with ensemble of regression trees were introduced in Kazemi and Sullivan [9] and in Ren et al. [10] using local binary features, reaching up to 3000 frames per second. Joint cascades of face detection and alignment improve the performance of both [41]. Robustness against large head pose variations or occlusions and self-occlusions due to large out-of-plane rotations have become a major objective to following studies with this approach [42,43,44]. By training on large face databases, DNNs improved regression performances, especially for large head rotations and 3D alignment [5,11,12,13,14,15,16,17,18,19,20,21,22,23,24,25,26,27,28,29,30,31], at the cost of much heavier computation than ensemble methods.

For video alignment, the performance of run face detection and image alignment run at every frame can be improved by exploiting spatio-temporal dependencies and temporal continuity. However, tracking without detection often suffers the drifting problem. A comprehensive comparison of detection versus tracking versus tracker-detector hybrids with failure detection, re-initialization and smoothness components are available in Cheysos et al. [45]. Cascaded regression methods require different sets of regression functions for detection and for tracking, since the level of initialization imprecision with detection is considerably higher. A spatio-temporal cascade is proposed with a time-series regression model in Yang et al. [46], and a cascade for incremental tracking is developed in Sanchez-Lozano et al. [47]. A joint optimization of alignment and tracking over a cascade with DNN-based features was proposed in Khan et al. [48]. Finally, end-to-end learning via DNNs was also applied to video, e.g., using recurrent neural networks [20,27] and two-stream transformer networks [24].

### 2.2. Event-Camera-Based Vision

Early EC studies focused on efficient low-level vision algorithms such as optical flow estimation, feature detection/tracking and motion segmentation [49]. They explored data representations and new frameworks for event-driven computing, needed to handle the unconventional event-stream and information encoding. More recent work focused on higher-level vision applications such as motion estimation and object recognition, using deep learning techniques. Due to the lack of large datasets, the proposed methods use pre-training on state-of-the-art networks of conventional image data and transfer learning using smaller EC datasets [50]. The collection of larger EC datasets shows improved performance of the proposed deep learning techniques over conventional sensing, mostly thanks to their robustness to challenging illumination conditions and fast motion [35].

An alternative approach to overcome the dataset handicap may be to perform video reconstruction [34].

A limited number of prior work is available on EC-based face processing tasks; no face alignment study exists to the best of our knowledge. For face detection, Barua et al. [51] applied a patched-based model via dictionary learning. Face tracking can work with a mass-spring system over parts of the face [52], or using eye blinks [53]. In Lagorce et al. [8], face recognition was tested with hierarchical time-surface representation of event-streams. Recovery of facial motion field and brightness while the camera is in motion were shown in several studies [34,54], where conventional cameras suffer from dynamic range limitations and motion blur. Finally, Savran et al. [6] applied spatio-temporal event-based convolution to locate and detect lip activity, and Li et al. [7] proposed a DNN on audio-visual events for speech recognition.

## 3. Face Pose Alignment Dataset

The absence of EC face databases is a major obstacle that hinders EC-based face alignment studies. While for alignment with conventional cameras huge databases were collected by downloading from the internet, the same approach is not possible with ECs. A temporary solution to the generation of large EC datasets is the use of video to events simulators [55,56], that interpolate the videos and simulate realistic noise conditions. This technique can be then supported by transfer learning techniques on real datasets. For this reason, we prepared a face pose alignment dataset, which is also included as a subset of an EC-based visual speech dataset [6]. The acquisition setup is presented in Section 3.1. We carefully prepared the content not only to include different subjects to increase appearance variability, but also to have a high degree of motion variability, as explained in Section 3.2. While EC datasets do not suffer from motion blur, this is true if the data visualisation adapts to the motion of the input. Long temporal windows create motion blur for fast moving targets, but short temporal windows can lack important information for slowly moving targets, as only a few events are generated and the full profile of the target can be incomplete. This poses a challenge for the manual annotation of datasets, required for the ground truth. To facilitate the annotation task, we developed an annotation tool described in Section 3.3 with which an annotator can aggregate temporal information through visualization to locate the facial features.

### 3.1. Acquisition

The database was acquired with the “ATIS” [2]. Since our goal was facial analysis, we aimed at close-up face shots, without background, that can be nevertheless added with data augmentation tools. Based on sensor resolution (304 × 240 pixels) and lens (f = 8 mm), subjects sit at about 70 cm away from the camera so that the face is covering a big part of the image plane. This setup results in 67 pixels frontal view eye-to-eye distance on average with 9.6 pixels standard deviation. As the average eye-to-eye distance is roughly 60 mm, 1 pixel corresponds to roughly 0.9 mm on average. The recording has standard illumination.

### 3.2. Content

In the dataset there are 108 clips of 10.2 min in total, from 18 subjects (9 males and 9 females). All of the subjects gave their informed consent before they participated in the study. The study was conducted in accordance with the Declaration of Helsinki, and the protocol was approved by the Ligurian Ethics Committee with identification CER_IIT_ECOMODE_01. Subjects were selected to include as many as visual variations as possible, comprising long hairs (which can partly occlude the face), different beard and moustache types. A different subset of eight subjects wear eyeglasses.

**Figure 1:**
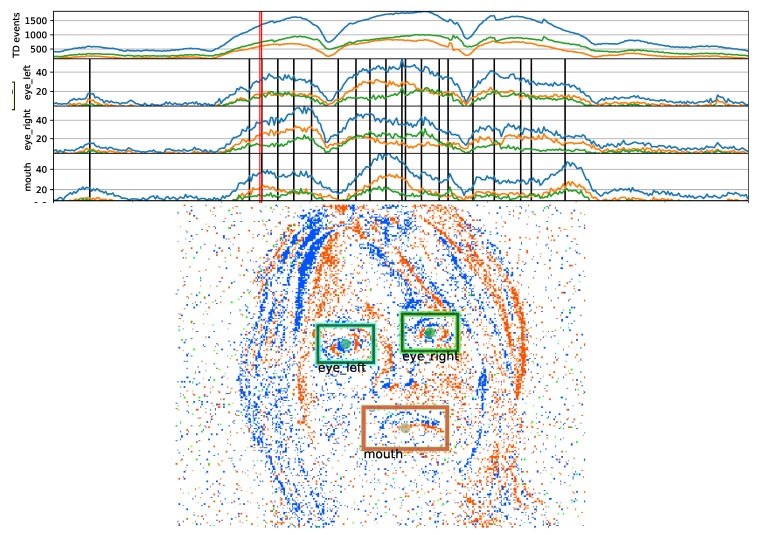
Snapshot of spatio-temporal eyes-mouth annotation of a rotating face, with a frame length and step size of 10 ms. Annotation times are shown with event rate sequences of rectangular areas displayed in the frame image (red thick line designates the frame image). Also the polarity events are plotted as time-series, and are colored differently in the frame image.

**Figure 2:**
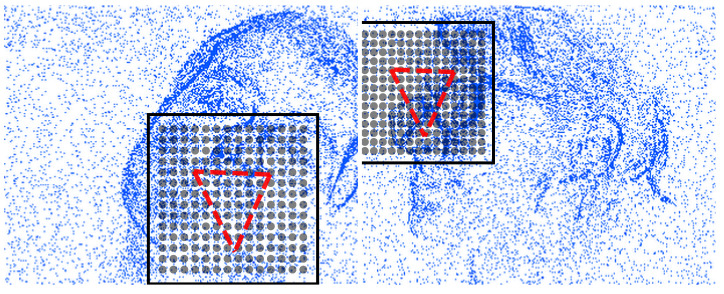
Two example superimpositions of motion detection grid (square domain and cell centers) as well as the reference eyes-mouth triangle according to the initial translations and scales.

Clips were collected during continuous head motion, and while subjects were talking and performing guided head movements. Since with EC datasets motion variability is a critical factor, we captured different motion types at varying speeds. We divide the clips into two categories, as intense and moderate head motion clips. The former category contains continuous roll, pitch and yaw rotations ranging from slow to very high speed. Clip duration, movement speed and number of head rotation repetitions vary arbitrarily from clip to clip. In the latter category, each subject turns his/her head in different directions starting from a neutral frontal face pose, then says a short sentence, and finally turns back to the starting pose. This category contains moderate head motion in terms of speed and angle. For each category, three clips were captured for each subject. The content of the clips is listed in Table 1, and various snapshots are shown in Figures 1, 2 and 4.

### 3.3. Pose Annotation on Event-Sensor Data

All the clips were annotated in a semi-automated way by human annotators marking eyes and mouth centers on pixel-event data. Figure 1 shows the visualization with the annotation tool developed for this task. The tool shows an image generated by accumulating events for 10 ms centered at a selected time-point. The pixel intensity is proportional to the event-rate, and the hue is proportional to the ratio of positive and negative pixel-events to gradually change the color. The tool also shows the event rate time profiles for the regions around eyes and mouth as well as for the whole image. The annotator can select a time-point where there is high event-count, i.e., when the face is visible thanks to relative movement with respect to the camera, then can visualize the corresponding image and click on the three reference landmarks (center of mouth, left and right eye) to generate the ground truth position. Event-rate profiles throughout the clip are estimated over the rectangular regions centered at the landmarks.

For all the time-points where there is no annotation, linear interpolation is applied to find the landmark coordinates. As soon as a landmark point is updated, the temporal plots are re-drawn by calculating event-rate for the interpolated landmark points as well. Since the peaks and high value regions in the event-rate plots correspond to significant motion, the annotator quickly finds where to annotate on the event-data, usually at the peak, onset and offset moments, and sometimes also at several in-between places if interpolated landmark points are clearly away from observed facial feature locations. Annotations usually start at high event-rate occurrences, then gradually more time-points are added and coordinates are refined by several passes over a clip.

Due to the ambiguity on landmark positions, annotation precision level is expected to be much lower compared to conventional image annotations. To evaluate annotation precision, we prepared two sets of annotations on the whole dataset with four annotators. In the first set, all the clips were annotated by the same person, and in the second, the dataset subjects were grouped into three and each was annotated by a different annotator. By calculating the disagreements between these two sets, using the standardized distance in Equation (10) at regularly re-sampled time-points as explained in Section 5.1, we found that the average disagreement error is 0.125 pixels with a standard deviation of 0.061. In all the experiments, we use the first set for training and testing, then compare the results with the second set, i.e., with inter-human annotation disagreements as a strong human-baseline.

## 4. Event-Based Face Pose Alignment via Cascaded Regression

In the proposed event-based method, face pose alignment is conveyed via a sequence of pose-events over a given temporal window. The input and output event sequences up to time *t* are $E_{t}=\left\{e^{i}\;|\;t^{i}\leq t\right\}$ where $e^{i}$ represents the pixel-event tuple indexed by *i*, and ${\hat{P}}_{t}=\left\{{\hat{P}}^{j},{\hat{t}}^{j},\;|\;{\hat{t}}^{j}\leq t\right\}$ where the predicted pose vector (${\hat{P}}^{j}$) and time-stamp (${\hat{t}}^{j}$) are indexed by *j*. A pose-event is generated when facial motion is detected, then alignment is performed by regression. In this study, we align for 2D affine pose, i.e., the relative pose of the face is an affine transformation of the template landmarks. Affine transformation has six degrees of freedom corresponding to 2D translation, scale, rotation, aspect ratio and shear, and is considered an approximation of rigid 3D pose on the image plane. Since motion of three non-collinear 2D points induces an affine transformation, we employ three landmarks to estimate the affine pose transformation of the face. Face pose is represented by the landmark coordinates vector $P={\left[x_{1},y_{1},x_{2},y_{2},x_{3},y_{3}\right]}^{T}\in R^{6}$. We use the eye and mouth center landmarks as in Dollár et al. [37]. As for the template, a mean shape in a standardized coordinate system, $\bar{P}$, is estimated over a training set by Generalized Procrustes Analysis (GPA) [57].

### 4.1. Event Sensor Data Representation in Space-Time

We convert the sensor’s pixel-events stream into a space-time representation to efficiently perform detection and alignment. EC sensors generate events asynchronously when logarithmic intensity changes at pixels are detected by adjustable thresholds [49]. Each pixel-event is represented by a tuple of values indexed with *i*, $e^{i}=\left(x^{i},y^{i},t^{i},p^{i}\right)$, where x,y is the location of the pixel in the image plane, *t* is the time at which the change has been detected, and *p* is a binary polarity value which represents the direction of the intensity change.

For down-scaling and efficient processing, events are down-sampled on regular space-time grids at multiple spatial scales. Cells in the grid are space-time volumes with square shape; for each location, there is a pair of cells of positive and negative polarities. Pixel-events falling in a cell at time *t* within the temporal window of length τ, $E_{t-\tau :t}$, are accumulated onto the cells of the multi-layered grid, G, where each cell is addressed by a multi-index tuple (p,s,i,j), which are of polarity (*p*), spatial scale (*s*), vertical (*i*) and horizontal (*j*) discrete coordinates. The set of the index tuples of G is the domain of the discrete function, $C_{t}$, which maps onto an event count, *C*:

(1) $$\begin{array}{c} C_{t}≜Accumulate\left(E_{t-\tau :t};G\right)\\ C=C_{t}\left[p,s,i,j\right]\;,\;C\in Z^{*}. \end{array}$$

$C_{t}$ can be imagined as spatial pyramids of positive and negative polarity of event-cell masses over the image plane, for a given temporal window at *t*. The resulting *event-mass* is an unnormalized down-scaled representation of an event sequence in multiple spatial scales. We set τ=40 ms as it obtains the best average alignment performance over various values from 10 ms to 160 ms. The step size of the time-frames is set to 20 ms for smooth transition.

### 4.2. Pose Motion Detector

We developed a motion detector to generate a *pose-event*, irrespective of the location of the face; i.e., the detector does not find where the face motion is but only when. Motion detection is based on pixel-event density over the face region, assuming big and frequent illumination change is very unlikely. We estimate local event-densities at different spatial scales separately and then average at each scale. This multi-scale density estimate is more informative than a single global estimate, since sizes of dense event regions depend both on the speed of motion as well as the scale. For this purpose, a multi-scale grid is created on the alignment template (in an offline phase). Then the density for each grid cell is obtained by re-sampling from the event-cell mass representation of the sensor output, $C_{t}$, defined in Equation (1), dividing event-masses by their cell areas. We perform nearest neighbor interpolation for scale-space re-sampling, as it is the simplest interpolation and finer details are not required for the detection. Density estimate is invariant to rotation and shape due to averaging over cells. Moreover, for invariance against motion polarity, we pick the maximum of the event polarities at each spatial cell coordinate, since an opposite motion direction inverts the event polarities.

The grid is generated by extending the square bounding box of an eyes-mouth template. To be able to cover the whole face region, its width is set to $3.5s_{rms}$ where $s_{rms}$ is the scale of the eyes-mouth triangle defined by Equation (10). Square grid cells are placed by overlapping half of their width and height. For a multi-scale grid with *L* levels, grid width for the scale *l* is

(2) $$\begin{array}{c} w_{l}=w_{min}{\left(\frac{w_{max}}{w_{min}}\right)}^{\frac{l}{L-1}} \end{array}$$

where $\left[w_{min},w_{max}\right]$ is the scale range. This provides *L* density estimates, hence features to be classified for detection. Spatial pyramids of event-cells are constructed in an octave range of ${\left\{sz.2^{-s}\right\}}_{s=1}^{3}$ where sz represent sensor width and height. We empirically set L=5 scales in the range $w_{min}=0.2$ and $w_{max}=1.0$. This multi-scale grid is superimposed on an initial pose prediction, $P^{0}$, to re-sample the event-densities. Superposition aligns only for the translation and scale due to invariance to rotation and shape. This alignment is done by finding the center and scale of the points in $P^{0}$. Scale is two times the radius which is given as the root mean square deviation from center. Two example detection grids (cell centers) at different scales with the superimposed reference triangular GPA shapes are shown in Figure 2.

Detection target (class label for presence of motion), y(t), for a time *t* and duration τ is calculated from the distance between two ground-truth pose vectors at the start and end of its time-frame as

(3) $$\begin{array}{c} y\left(t\right)=I\left(∥P_{\left(t+\tau /2\right)}-P_{\left(t-\tau /2\right)}{∥}_{2}>0\right) \end{array}$$

where I is the indicator function (returning 1 if the logical proposition holds, or 0 otherwise). $P_{\left(t\right)}$ is estimated by linear interpolation on ground-truth labels which are assigned to key frames over a clip via manual annotation. To predict we use logistic regression with $L_{2}$ regularization and by class weighting to balance the unbalanced positive and negative sample sizes. Detector threshold is determined on a validation set to meet a certain false negative rate (FNR) as explained in Section 5.1.

### 4.3. Pose-Invariant Cascaded Regression

The key factor that leads to high performance alignment with the cascaded regression framework is the pose/shape invariance integration to gradient boosting machines (reviewed in Section 2.1). With a cascade of length *K*, pose prediction is recursively performed by

(4) $$\begin{array}{cc} {\hat{P}}^{k} & =r^{k}\left({\hat{P}}^{0}\right)\\ \; & =r^{k-1}\left({\hat{P}}^{0}\right)+{\left(M_{S}^{k-1}\right)}^{-1}\circ g^{k}\left({\hat{P}}^{k-1}\right) \end{array}$$

where k∈1,…,K, $r^{k}\left(.\right)$ is the partial cascade regressor of length *k*, ${\hat{P}}^{K}=r^{K}\left({\hat{P}}^{0}\right)$ being the output of the complete cascade given a starting point, ${\hat{P}}^{0}$, and with ${\hat{P}}^{0}=r^{0}\left({\hat{P}}^{0}\right)$ as the initial prediction. $g^{k}\left(.\right)$ is the stage regressor at stage *k* which predicts a displacement vector to approach the true pose coordinates, however, in a normalized coordinate system. Please note that the regressor operates on pixel-event cell representation (Equation (1)), though we omit here for brevity. ${\left(M_{S}^{k-1}\right)}^{-1}$ is the similarity transformation which maps the displacements predicted by $g^{k}\left(.\right)$ in a normalized coordinate system back onto the observation domain (here, ∘ denotes rotation and scale only transformation since translation has no effect on displacement). Normalization is realized by mapping onto a template, which here is the mean shape obtained by GPA. Training targets of $g^{k}\left(.\right)$ are the normalized residual displacements,

(5) $$\begin{array}{c} \Delta_{S}P^{k}=M_{S}^{k-1}\circ \left(P-P^{k-1}\right), \end{array}$$

where *S* denotes the similarity normalization by $M_{S}^{k-1}$ which is estimated via linear least square minimization

(6) $$\begin{array}{c} M_{S}^{k-1}=\underset{M_{S}}{\arg\min}{∥\bar{P}-M_{S}\cdot {\hat{P}}^{k-1}∥}^{2}. \end{array}$$

The same minimization is also used in prediction to directly estimate ${\left(M_{S}^{k-1}\right)}^{-1}$ for the opposite mapping. Thus, the training set for $g^{k}\left(.\right)$ is composed of triplets ${\left\{\left(E_{i},P_{i}^{k-1},\Delta_{S}P_{i}^{k}\right)\right\}}_{i=1}^{N}$, where $E_{i}$ represents the pixel-event observations for the ith sample.

Pose/shape invariance is gained by re-sampling the features at each stage *k* relative to the last pose/shape estimate ${\hat{P}}^{k-1}$, through a map $M_{P}^{k-1}$ such that $\bar{P}=M_{P}^{k-1}\circ {\hat{P}}^{k-1}$. Though the attained invariance is approximate as shown in Dollár et al. [37], in practice it works well since the prediction at each stage becomes more aligned with the true landmark coordinates. Within the cascaded regression framework, instead of warping the observations with $M_{P}^{k-1}$ to extract pose-invariant features, feature coordinates in the standardized template coordinate system are mapped onto the observation domain via the inverse warp ${\left(M_{P}^{k-1}\right)}^{-1}$ for re-sampling. This is because transforming only a few coordinates is computationally much simpler, as a sparse feature coordinate set is sufficient for good prediction in this framework. Thus, given a set of standardized feature locations, $\left\{x_{i}\right\}$, the re-sampling coordinates are obtained via

(7) $$\begin{array}{c} x^{\prime }={\left(M_{P}^{k-1}\right)}^{-1}\circ x. \end{array}$$

We apply Equation (7) by the computationally lightweight approximation suggested in Cao et al. [40], which avoids direct estimation of ${\left(M_{P}^{k-1}\right)}^{-1}$ to simplify calculations.

Except the initial work by Dollár et al. [37] where weak regression is performed at each cascade stage using random ferns, strong regression is commonly employed for the stages. Similar to Cao et al. [40], we found that boosting of strong regressors provides faster convergence in training and higher performance in testing, confirming their conjecture also with an EC, i.e., weak learners cause unstable pose-aligned feature coordinates. We apply bottom-level gradient boosting without pose/shape invariance updates as the strong stage regressor of the top-level, as in Kazemi and Sullivan [9], Cao et al. [40] and Lee et al. [44]. Also we use regression trees instead of ferns [40] as they offer high performance when applied as random forest or extremely randomized tree (ERT) ensembles. More specifically, we apply ERT similar to Kazemi and Sullivan [9], however, with a model for pixel-event masses as explained in Section 4.4.

### 4.4. Extremely Randomized Trees over Event Masses

We use extremely randomized trees [58] with event-cell mass pair difference features to predict normalized residual pose displacements. These features are similar to pixel difference features which are commonly preferred due to very lightweight computations while providing high capacity with regression trees [9] or ferns [37,40]. As the leaves of trees or ferns constitute some linear combination of training samples, face shape constraint is satisfied intrinsically for alignment. In order to have invariance to motion direction when aligning over pixel-event data, we take the maximum over polarity masses at each cell location. ERT fitting is completely stochastic including feature selection, i.e., by randomly sampling features as well as decision functions. Due to the infeasibly high dimension of all possible location pairs, candidate features are randomly sampled but computed only when needed for a random test that is performed for each split node of each tree. Binary test for a decision function with a pair difference feature is defined as

(8) $$\begin{array}{cc} b(C;{\hat{P}}^{k-1})= & \left\{\begin{array}{cc} 1 & C_{max}\left(y_{1}^{\prime },x_{1}^{\prime }\right)-C_{max}\left(y_{0}^{\prime },x_{0}^{\prime }\right)>c_{th}\\ 0 & \mathrm{otherwise} \end{array}\right.\\ \; & C_{max}\left(y,x\right)=\max_{p}\left(C(y,x,p)\right) \end{array}$$

which uses the threshold, $c_{th}$, while testing the difference of a maximum polarity cell mass pair, i.e., a pair of $C_{max}$. If the evaluation result is one, the left child, otherwise the right child node is selected. Here, $x_{n}^{\prime }={\left[x_{n}^{\prime },y_{n}^{\prime }\right]}^{T}$ are the coordinates on the input domain which are obtained by mapping the pose-indexed feature coordinates from the template domain onto the input via Equation (7). We perform nearest neighbor re-sampling on the base level of the event-cell mass pyramid (see Equation (1)). By sampling many random pairs and decision thresholds, a set of candidate decision functions are created for a node, and evaluated with the total square error loss over the training set which is assigned to that node. Feature coordinates are initially sampled in the template domain with a uniform distribution over a square region encapsulating the template (to speed up training a sufficiently large random pool of coordinates is sampled together with the event-masses at the beginning of each pose-invariant stage). However, to focus on the local patterns of facial features, we re-sample using the prior proposed in Kazemi and Sullivan [9]

(9) $$\begin{array}{c} p\left(x_{0},x_{1}\right)\propto e^{-\lambda ∥x_{1}-x_{0}{∥}_{2}} \end{array}$$

setting λ to match local feature scales in the standardized domain. The decision thresholds are sampled with a bounded uniform distribution where the boundaries are set according to the typical range of pixel-event masses. Then learning at a split-node is realized by selecting the candidate combination which yields the minimum training error.

### 4.5. Clustering-Based Multiple Initialization

We apply clustering-based multiple initialization, to improve learning as well as prediction. In training, it simply serves as a data augmentation technique to increase the generalization capability. In prediction, each different initial pose leads regression to a different alignment, which helps to improve robustness by reducing the risk of misalignment, as commonly employed in the regression framework. It is especially crucial in our study due to highly varied and ambiguous differential EC observations as well as due to big head rotations.

In the training phase, we aim at finding a diverse set of pose perturbations to be added to a given initial pose, $P^{0}$, which also have a high occurrence probability given a training set. For this purpose, we apply the k-means algorithm on the set of normalized displacements ${\left\{\Delta_{S}P_{i}\right\}}_{i=1}^{N}$. Having *C* cluster centers of normalized translations, ${\left\{\Delta_{S}P_{c}\right\}}_{c=1}^{C}$, multiple initialization are calculated by $P_{c}^{0}=P^{0}+{M^{0}}_{S}^{-1}\circ \Delta_{S}P_{c}$.

As the resulting pose vectors after multiple regression can be widely distributed, mean estimate over them usually causes inaccurate alignments. Therefore several methods were applied in the literature. Among them are pose clustering to get the mode of the most dense region [37], component median [40], and optimizing via replicator dynamics [42]. In this work we use the component median since its computation is extremely simpler, while being satisfactorily accurate for the task.

## 5. Experimental Results

We first describe various components of our evaluation methodology in Section 5.1. Then in Section 5.2, we compare the prediction performance of our method with a second set of human annotations, i.e., against a human-baseline. Finally, we analyze the effects of face resolution in Section 5.3 and model complexity in Section 5.4.

### 5.1. Evaluation Methodology

**Pose-event rate:** We measure the output pose-event rate in Hz as $f_{det}=\hat{P}/T_{total}$, where $|\hat{P}|$ and $T_{total}$ are the total number of predicted pose events and total duration, respectively.

**Error metric:** Alignment quality is evaluated via scale-invariant landmark localization error, as average Euclidean distance divided by a scale estimate. To account for large pose variations, scale estimates are based on bounding boxes of landmarks, either calculating the box diagonal [45] or the geometric mean of width and height [5], since the commonly used inter-ocular distance underestimates the scale with yaw. For more accurate scale estimates under large poses, we calculate the root mean square (RMS) of the three landmark deviations from center. Thus, the pose alignment error is

(10) $$\begin{array}{c} E_{P}=\frac{1}{3}\frac{\sum_{k=1}^{3}{∥{\hat{p}}_{k}-p_{k}∥}_{2}}{s_{rms}},\;s_{rms}=\sqrt{\frac{\sum_{k=1}^{3}∥p_{k}-\bar{p}{|∥}_{2}^{2}}{3}} \end{array}$$

where $\bar{p}$ is the center of the ground-truth landmarks, and $p_{k}$ and ${\hat{p}}_{k}$ are the ground-truth and predicted coordinates for the kth landmark, respectively.

**Accuracy and precision:** We evaluate accuracy and precision using the error metric in Equation (10). Accuracy is measured by a failure percentage. We assume that, within a maximum error allowed for successful alignment, human annotations of the same pose should agree with 99% probability. Therefore the failure threshold is set to the 99th percentile of the annotator disagreement error, which is 0.308. As a consequence, precision is calculated as the average error of the predictions that are deemed as successful.

**Regular temporal re-sampling:** Due to sparse prediction, we perform regular re-sampling via bounded nearest neighbor (BNN) interpolation since each event has a finite time-support. This is realized by assigning the index of the predicted pose-change event at *t* according to

(11) $$\begin{array}{c} j_{BNN}\left(t\right)=\underset{j\in \left\{d_{j}\left(t\right)\leq \frac{\tau }{2}\right\}}{\arg\min}\left[d_{j}\left(t\right)\right]\;,\;d_{j}\left(t\right)=t-{\hat{t}}_{j}. \end{array}$$

Thus, if the normalized distance is $d_{j}\left(t\right)>\tau /2$, then $j_{BNN}\left(t\right)=\emptyset$, meaning time-point *t* is not supported by any pose-event, i.e., alignment is not activated. For those time-points, we take the given initial pose as the prediction not to cause a misleading bias on error statistics. The re-sampling step size is 1 ms. For the regular re-sampling of the ground-truth that is available only at key-frames, linear interpolation is applied.

**Speed-balanced evaluation:** With ECs, motion is a major source of variation unlike in the conventional camera observations. Especially head movement speed is a crucial variable that can affect performance. Highly imbalanced speed distribution of a test dataset can be misleading when generalized for dissimilar speed distributions. Therefore it is essential to assess speed dependent alignment performance. For this evaluation, we do speed quantization by partitioning the regularly re-sampled test samples according to their ground-truth speed levels. Moreover, we can practically estimate expectations over any distribution of the speed levels. Particularly in this study, we take expectation on the uniform speed distribution to be able to evaluate with a more generalized average performance score, i.e., to suppress the speed bias of the test set. We calculate the observed speed on the image plane by estimating RMS speed of the pose vector (concatenated landmark coordinates) at time-point *t* by differentiation

(12) $$\begin{array}{c} v_{t}=\frac{d_{t}}{\Delta t}=\frac{∥P_{\left(t+\Delta t/2\right)}-P_{\left(t-\Delta t/2\right)}{∥}_{2}}{\Delta t\sqrt{3}} \end{array}$$

where $d_{t}$ is the RMS distance between successive ground-truth pose vectors. Then we compose six speed levels with the upper interval boundaries: {1,75,150,225,300,375,450} pix/s (pixels per second). The range of [0,1] pix/s is evaluated separately, by labeling them as still head poses. We chose six speed levels; because, while it allows us to observe sufficiently different levels of movement speeds, it is also small enough to concisely report the evaluations. The upper limit is chosen to exclude very high speed outliers; after a statistical outlier analysis and then rounding to 450 for convenience as it is divisible by six.

**Dual cross validation:** We evaluate by cross validation and by separating training and test subjects, since the subject variation is limited by 18 subjects in our dataset. As we have two different predictors running in a cascade, their training and testing are performed by two different cross validation. For the detector, we apply 3-fold cross validation providing (12,6) splits of the subjects; and for the pose regression, 6-fold cross validation is applied providing splits of (15,3). We opted for a smaller training set for the former to considerably reduce the training and testing times as the method requires multiple passes over the whole EC database. Because the model complexity of the detector is very low, i.e., there are only a few parameters to estimate, smaller training sets do not deteriorate the detection performance. Also, the training set of the detector is further divided as (8,4) to adjust the FNRs on four subjects within each training fold while fitting on the eight. We set the FNR goal to 0.05 as it provides a good compromise between detection and pose-event rates, and observed that by this method resulting FNR values on the test sets met the goal closely.

**Initialization:** In a complete face analysis system, a face template is initially aligned via a coarse similarity transformation estimate. Commonly the face detection bounding box is used to obtain coarse location and scale estimates. A better initialization can be made in a tracking framework based on temporal continuity of pose/shape vectors [45], with joint detection and alignment [41], or using a coarse pose estimation [46]. Good initialization simplifies the alignment task. However, regression-based methods can be sensitive to the face detector as well as the bounding box configuration, since a model of mapping learned in the training stage may not correspond well to the mappings due to initialization with a different detector applied in testing in Sagonas et al. [59]. For experimentation, we simulate face detectors of different quality regarding the position and scale uncertainty, with the expectation that a typical detector predicts the face position within the size of the eyes-mouth triangle. We assume rotation and the rest of the affine pose components are totally unknown, therefore we fix them to identity transformations. For this reason, in-plane and out-of-plane rotations can cause very high errors even when translation and scale initialization is good. Translation is sampled using a uniform density over a circular region of radius $r_{trl}$, and scale is sampled within a uniform density at logarithmic scale of the range $\left[1/r_{scl},r_{scl}\right]$. Since $s_{rms}$ in Equation (10) is the statistical size measure, we parameterize the translation range in the units of $s_{rms}$. We experiment with three levels of scale-space localization uncertainty settings given in Table 2. While the low simulation setting can be considered to be a state-of-the-art face detector with conventional cameras or a tracker with significant jitter noise, the medium and higher cases correspond to less precise detectors. We simulate also by high localization imprecision since EC-only face detectors may be much less precise than the conventional face detectors due to differential observations and motion induced uncertainty.

### 5.2. Comparison against Human Performance

Since there is no prior work on pose alignment with ECs, we evaluate our method by comparing against a second set of human annotators who annotate the landmarks on the EC observations as described in Section 3.3. Thus, the errors due to the disagreement between the two sets of annotators serve as a very strong EC baseline.

For feature search and selection in the training for regression, we sample event-cell pairs over a square area of width $8s_{rms}$ centered at the input pose center; because, relatively large sampling domain is helpful to capture patterns that can occur far away due to motion or due to poor initialization. We apply down-sampling to lower the spatial input resolution as explained in Section 5.3. We empirically found good parameter values of feature selection that help to complete training in a reasonable amount of time without degrading the performance significantly as follows. The prior on the coordinate pairs is modeled by λ=0.5 with the $1/s_{rms}$ unit (Equation (9)). The random feature selection pool size is set to 1000. We sample cell-mass difference thresholds of the node splits, i.e., $c_{th}$ in Equation (8), in the range of $\left[-0.01,0.01\right]\cdot \tau \cdot \Delta x^{s}\cdot \Delta y^{s}$ events, i.e., within the centered density interval of 0.02 events/(ms · pix^2^). 40 trials are performed to find an optimal pair of features and decision thresholds at each tree node.

We adjust the predictor model with an optimal complexity as follows, which is comparatively evaluated in Section 5.4. A forest of 40 trees at each stage of gradient boosting is used, as it is sufficiently big to regularize while small enough not to degrade the accuracy. Tree depth is set to four. 16 initialization clusters are estimated to fit the model as well as to be used in prediction. Both the bottom-level and top-level cascade lengths are set to 10, hence feature sampling for pose-invariance is done at every 10 stages and the total cascade length is 100.

Table 3 compares our predictor performance and inter-human annotation disagreements by average accuracy and precision in terms of failure percentages and precision errors, separately for intense and moderate head motion clips as well. The averages are taken excluding the still frames that are in RMS speed range of [0,1) pix/s. Here the predictor runs under low initial localization uncertainty setting (see Table 2). We see that inter-human disagreements in terms of average accuracy and precision are smaller than prediction errors, as expected. However, for moderate head motion clips, average prediction errors are very close to inter-human disagreements.

Human superiority is seen for almost all the head movement speed levels in Table 4 as well. The only exceptions are the second fastest level ([300, 375) pix/s) and the speed levels above 75 pix/s in the moderate motion category where the results are comparable. In general, at the fastest and slowest movements alignment mismatches are getting worse. While inter-human disagreements are slightly increasing, predictor performance worsens strikingly, especially at the highest speed level. These degradations can possibly be due to higher degrees of ambiguity, as in a fixed temporal window significant motion blur can occur at the fastest speeds and observations can become very weak at the slowest speeds.

We also see the resulting pose-event rates in Table 3. EC skips still head pose moments by means of motion detection, thus avoids unnecessary processing for alignment. The resulting average pose-event rate is 32.2 Hz as seen in Table 3. The processing rate with conventional video frames corresponds to the fixed rate of 50 Hz due to 20 ms time-steps. Please note that pose-event rate is even less with moderate motion clips.

**Figure 3:**
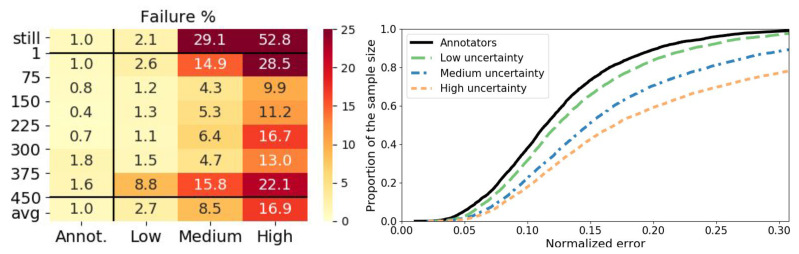
Comparison of prediction and human annotation under low, medium and high localization uncertainty ranges. Failure percentages are shown on the heat map where rows are the speed levels (still: no head motion label [0,1) pix/s, avg: average over levels excluding [0,1) pix/s). Cumulative distribution curves of the precision error are shown on the right where speed balancing is realized by sub-sampling without replacement equally from each speed interval.

Face detectors or trackers provide face position and scale with some degree of uncertainty to initialize the alignment. To characterize with respect to scale-space localization uncertainty, and thus to be able to determine required precision levels of EC-based face detectors and trackers, we evaluate under three uncertainty ranges given in Table 2. Figure 3 compares the accuracy and precision of our predictor and human annotators (using an optimal model complexity configuration for each uncertainty level according to Section 5.4). Please note that the resulting average pose-event rates for the three uncertainty levels are almost the same; as 32.2 Hz, 32.0 Hz, 32.7 Hz from low to high uncertainty, respectively. On the left of Figure 3, the heat map shows the failure percentages of the individual speed levels as well as the averages over all excluding the still head labels. As expected, failures escalate with increasing uncertainty range for all the speed levels. The worsening effect at the fastest and slowest motion levels as explained above are much more prominent with increasing localization uncertainty. Moreover, the two slowest speed levels cause more severe degradations, which can be explained by undetected motion not activating the alignment, since in the absence of activity EC sensors only emit pixel-events due to noise.

On the right of Figure 3, cumulative distributions of the precision error are shown, after speed level balancing by sub-sampling without replacement from each speed interval with the size of the smallest speed partition. For all the uncertainty levels, the curves start from similarly low error values, but with increasing error, higher uncertainty in localization makes the error distribution more dissimilar to the annotators curve worsening the precision.

**Figure 4:**
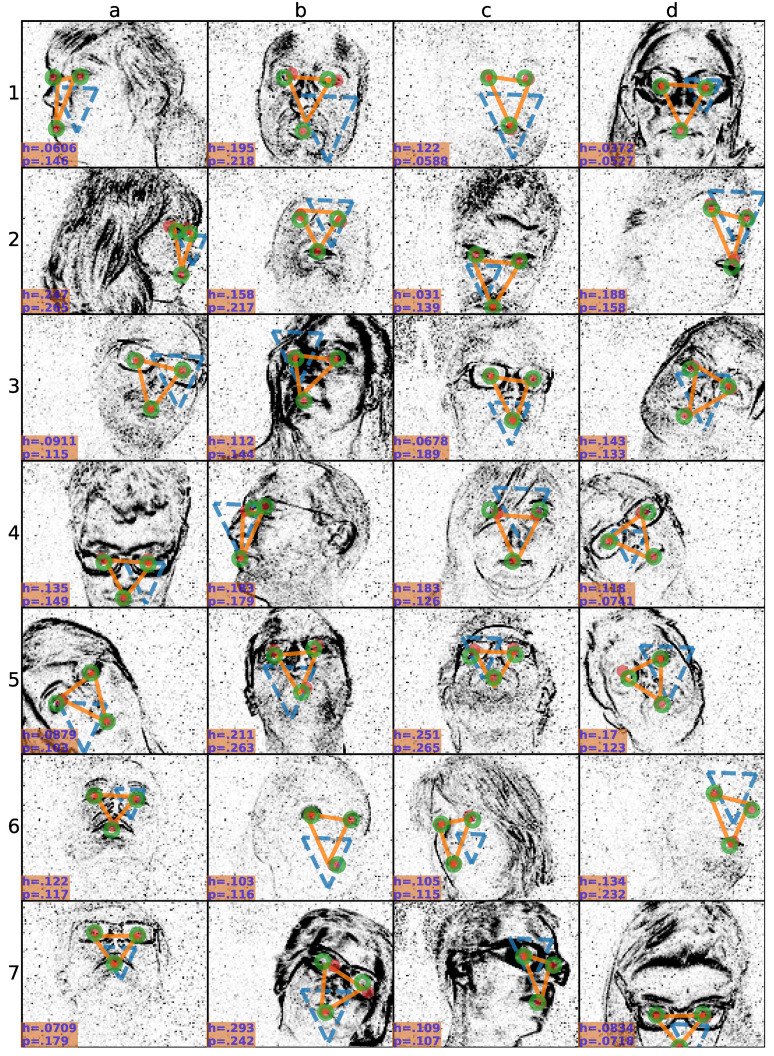
Snapshots of alignments at the high localization uncertainty setting. Each plot (**a**–**d**, **1**–**7**) shows a different snapshot from a different subject, or head pose. At each snapshot ground-truth annotation (red dots), second human annotation (green circles), initialization (blue dashed triangular shape), and prediction (orange triangular shape) are overlaid. Inter-human disagreement (h) and prediction (p) errors are written at the left-bottom corners.

Figure 4 shows example snapshots of alignments (orange triangular shapes) at the high initial localization uncertainty setting (initialized by blue dashed triangular shapes) compared to the two sets of human annotators (ground-truth: red dots, second set: green circles). We see accurate prediction of landmarks (below the failure threshold 0.308) for various poses involving talking lips and eye blinks as well as roll, yaw and pitch rotations. An example of alignment by eye blinks are at the cell “6b”, and by talking lips are at “1c”, “2b”, “2d”, and “6d”. Alignment can accurately be done also when the mouth is cropped as seen at “7d”. Precision of the detector, in general, slightly worse than the human annotators. However, occasionally there are opposite cases as well. For instance, due to the hair occlusion on the left eye at the cell “4c”, human disagreement is higher than the prediction error whereas prediction could locate the left eye similarly to the ground-truth. Small annotator disagreements are observed also due the talking lips in “2d” and on the eyes due to a fast movement at “7b”.

### 5.3. Evaluation of Face Resolution

In our dataset, face resolution is quite high, as eye-to-eye distance frontal view is 67 pixels on average. Depending on the sensor, lens, and distance to camera, acquired face resolution can be much lower in different applications. Lower resolutions might have an unexpected negative impact on the performance, since event sensor data has the complexity of high degree of variations due to motion dependence which may complicate the alignment if some necessary details are lost. Conversely it may also lead to improvements, since learning is facilitated due to reduced feature search space which originally contains too much redundancy for the alignment task. To clarify these possible cases, we investigate the effects of lower resolutions on the performance.

**Figure 5:**
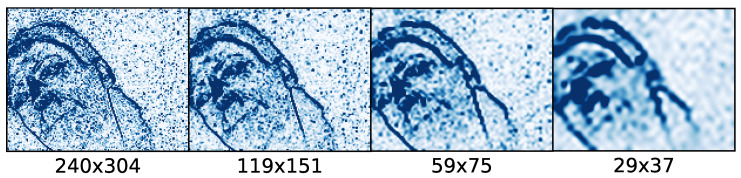
Pixel-event snapshots of 40 ms frame at different spatial scales while head is rotating (71.2 pix/s RMS speed). The scales from left to right are 1, $2^{-1}$, $2^{-2}$ and $2^{-3}$, with pixel sizes written below.

We experiment by applying spatial down-sampling via pixel-event cells, with the down-scaling ratios of $2^{-1}$, $2^{-2}$ and $2^{-3}$, and using odd cell sizes. Example snapshots of the resulting spatial-scales are shown in Figure 5. Table 5 shows the corresponding event-cell sizes, resolutions and alignment failure percentages for the three levels of uncertainty depending on the down-sampling ratio. Failures increase one octave below $2^{-3}$ for all the uncertainty levels. In this experiment, to reduce the experimentation time, we use four initialization clusters and tree depth of four. It is seen that optimal down-sampling ratio also greatly depends on the localization uncertainty. The optimal ratios are $2^{-2}$ for the low level, and $2^{-3}$ for the medium and high level uncertainties. These results indicate the benefit of eliminating redundancy and fine details for the coarse pose alignment task with ECs, especially for the more challenging cases of higher localization uncertainties, as well as demonstrate the pose alignment capability for wider range of applications and with cheaper lower resolution sensors.

### 5.4. Evaluation of Model Complexity

Complexity of a predictor alters the trade-off between computation requirements and performance. Also, higher complexity models can cause over-learning which degrades the generalization on the test set. For these reasons, we analyze the performance of EC-based pose alignment by varying the complexity of our predictor, for different levels of localization uncertainty ranges. The main parameters that change the model complexity are tree depth, cascade length and number of initialization clusters. We observed significant improvements up to 16 initialization clusters. For instance, more than 3% of failures are recovered by increasing the clusters from four to 16 for the high uncertainty level case, and about 1% for the other ranges. Higher number of clusters improves the performance by reducing the chance of getting stuck at poorly initialized predictions and by enabling a richer data augmentation in training. However, from 16 to 32 the improvements were negligible with the expense of doubling the computations. Therefore we set the number of clusters to 16.

Second, we investigate the effects of tree depth. Table 6 shows that increasing the tree depth improves the learning capacity, up to a certain level after which over-fitting starts to cause significant degradation. While depth of six is the best for the high and medium uncertainty cases, four levels obtains the lowest rate for the low case.

**Figure 6:**
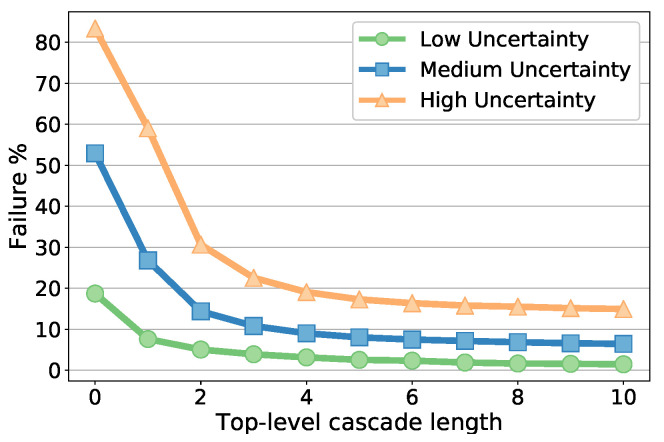
Failure percentages with varying cascade length for the three initialization uncertainty ranges.

Finally, we evaluate the cascade length in Figure 6 which shows the failure percentage versus top-level cascade length, for each localization uncertainty level using the best tree depths according to Table 6. The majority of the improvements are rapidly achieved in the first few stages, and then failure percentages slowly decrease. We picked the length of 10 for all the comparative evaluations in this paper, though some small amount of improvement is still possible with longer cascades. Similarly, the length of the bottom-level cascade is also fixed at 10 (hence total number of stages is 100), due to insignificant changes on the results. The results with the selected optimal parameters are reported in Table 7.

## 6. Time Complexity Analysis

We estimate time-complexity of the proposed face pose alignment as a measure of its efficiency. It depends on pose-event rate and on the time-complexity of the cascaded regression. Compared to regression, time-complexity of pixel-event accumulation (integer increments) and of the detector are negligible. While pixel-events are accumulated at a rate depending on scene activity and sensor configuration, the motion detector has a fixed rate determined by the time-step, and each detection activates the pose regression, thus generates a pose-event.

The time-complexity of the detector is $O(N_{det})$ where $N_{det}$ is the total number of cells in the multi-scale grid of *L* levels. For each cell, superimposition (a multiplication and an addition) and nearest neighbor re-sampling operations are performed. As operations per second, i.e., in Hz, time-complexity is measured by the product of the detector sampling rate and complexity, $O_{Hz}\left(fN_{det}\right)$, where *f* is the sampling rate. Since 20 ms time-steps correspond to 50Hz rate and $N_{det}=1886$ (for multi-scale grid of L=5 levels), scalar multiplication operations per second is expressed as $O_{Hz}\left(50\times 1886\right)=O_{Hz}\left(94,300\right)$. It is much lower than of the matrix operations for alignment which is given in the sequel. Therefore, the complexity of the detector becomes dominant only for the rare or no activity cases. It can also be reduced by tuning for the spatial grid resolution and detection trade-off (being already lightweight, we did not tune in the experiments).

Time-complexity of a single pose-invariant cascaded regression is determined by the alignment refinement performed at each top-level cascade stage since it involves matrix multiplication of feature coordinates, as the most expensive operation. The maximum number of matrix multiplications is twice the total number of split nodes, since there is a feature point pair at each. However, since we sample from a random coordinate pool at each top-level stage (see Section 4.4) which has a total point size less than of the number of the feature points in the ensemble due to the parameter configuration in Section 5.2, the upper bound in our study is lower. Given $N_{pool}$ pool feature points per stage and $N_{mult}$ multiple regressions, the complexity is $O\left(N_{align}\right)=O\left(N_{mult}\cdot K\cdot N_{pool}\right)$. The sum over six dimensional pose displacement vectors of the tree leaves is relatively insignificant. On the other hand, the overall detection-based pose alignment complexity depends on the detection frequency, $f_{det}$, which in turn changes depending on the input, i.e., scene activity. The upper bound on the operations per second is $O_{Hz}\left(f_{det}N_{align}\right)$.

Since the average detection rate on the whole dataset is $f_{det}=32.2$ Hz (see Section 5.2), applying the alignment complexity based on upper bound on the matrix multiplications with $N_{mult}=16,K=10$, $N_{pool}=1000$, the operation rate is obtained as $O_{Hz}\left(32.2\times 16\times 10\times 1000\right)=O_{Hz}\left(5,152,000\right)$. Also, after reducing by $N_{mult}=4$ for the low uncertainty setting with the expense of less then 1% drop on performance, we obtain $O_{Hz}\left(1,288,000\right)$. In MFlops (mega floating point operations per second), these values translate to about 5 MFlops and 21 MFlops, as total scalar multiplications due to 2×2 matrices. The complexity of the detection is insignificant compared to the matrix multiplications performed in alignment. Moreover, we also observed that by reducing the pool size, $N_{pool}$, the regression computations can be greatly reduced with only small alignment performance loss. However, in the absence of significant activity, operation rate will be at the bottom levels of the motion detector. On the other hand, the same alignment with the same time-steps would be at a fixed video rate of 50 Hz, hence with about 1.6 times more operations.

Similar cascaded ensemble regression-based methods on video are the fastest methods in the literature (1k frame per second [9] and 3 kfps [10]). While computation speed is not a measure of efficiency, since speed depends on other factors and usually relies on power-hungry hardware acceleration, methods that perform at such high speed can trade off high efficiency with speed, similarly to implementations based on pose-event rate. Real-time DNN inference is a challenge, and faster DNNs can only be obtained by means of binarization [26]. Efficiency of DNNs is by no means comparable to the efficiency of cascaded ensemble based prediction as they require GPUs which have demanding power requirements. While our method only performs coarse alignment with three landmarks, prior methods perform detailed alignment with about 70 [10] or 200 landmarks [9]. Although there is no linear relationship between landmark count and efficiency, still low landmark count considerably improves efficiency. Combining with the efficiency of the sensor (we elaborate on it in Section 7), we can safely conclude that the proposed face pose alignment is an extremely efficient method to fulfill the coarse alignment task we aim at.

## 7. Discussion

Compared to frame-based image acquisition, bio-inspired, activity dependent, sparse encoding from ECs offers lower power consumption, higher temporal resolution, higher dynamic range, at the cost of lower spatial resolution and lack of full image intensity measurements. The continuous improvement of such sensors in terms of spatial resolution, quality and cost, thanks to the involvement of companies that are developing products for mass production, and the demonstration that their output is highly informative [60,61], support the development of algorithms such as the one proposed in this work. Below we discuss all these pros and cons in the context of EC-based face pose alignment.

**Low power consumption.** EC power consumption inversely depends on scene activity, and power requirements stay at minimal levels in the absence of motion. For instance, ATIS sensor [2] that is used in this work consumes 50 mW in the idle state while 175 mW with intense scene activity. The values for some other well known ECs changes as follows: 27–50 mW for Samsung-DVS-Gen2 [62], 10–170 mW for DAVIS346, 5–14 mW for DAVIS240 [1] and 23 mW for DVS128 [63]. Facial movements elicit a low number of events, as they are produced only during head motion, or blinks and speech production, maintaining the sensor’s activity, and hence power, at a minimum. In comparison, power consumption of conventional cameras mounted in mobile phones are around 1 W and can go up to 1.5 W depending on the model [64]. Therefore ECs are highly suitable in battery critical scenarios. Such cases are specifically common in robotics and for some mobile device applications. For instance, as a mobile phone application, we can use our method either for pose-free or pose-dependent extension of voice activity detection which has shown to be power efficient and noise robust [6]. As the phone stays active for long durations, EC-based processing can save considerable energy, and with the advantage of using the visual channel to cope with noisy acoustic environments. Other types of facial actions, head/facial gestures and expressions, can also be implemented similarly for low-power scenarios by employing coarse pose alignment. Thanks to the adaptive sampling, ECs can considerably reduce computation as well, if the rate of processing is adapted to the pixel-event rate. In the pipeline proposed in this paper, the average processing rate ranges between 29.1 and 36.3 Hz (for clips with moderate and intense motion, respectively), roughly 40% less than in a frame-based implementation (Table 3).

**High temporal resolution:** ECs can provide up to MHz sampling resolution with high speed input motion, and with proportionally low latency. The stream of events from ECs does not suffer from motion blur [54], which is often observed in images of fast head rotations, on the mouth during speech, or due to camera motion, avoiding the need to implement costly de-blurring in face alignment [12]. ECs are therefore suitable in applications where motion provide the most relevant information, as in facial action recognition, voice activity detection and visual speech recognition, that must be robust to face pose variations.

**High dynamic range:** Although exploitation of the high intra-scene dynamic range feature of ECs is out of scope of our study, in general, it offers robustness to changing and uneven illumination conditions in all applications in uncontrolled environments, as shown for facial image reconstruction [54], for autonomous driving [35] and for drone vision [65].

**Lack of full intensity measurements:** Conventional methods work on whole static intensity images, and therefore their precision and detail level (number of landmarks) can be very high. Intensity reconstruction may be considered to be an alternative way for higher precision alignments but it requires expensive computation. For these reasons, certain kinds of applications are not suitable, at least currently, with ECs. For instance, facial performance capture for animation or 3D reconstruction of the whole facial surface and applications based on it (3D face recognition, face transfer, etc.) can be realized by using video cameras with much less effort and with higher fidelity.

**Low spatial resolution:** Most of the current ECs have lower spatial resolutions than video cameras. Nevertheless, in various applications where motion and dynamics information are the most relevant, high spatial resolution is not critical, unlike texture related processing, such as in 3D face surface reconstruction. Besides, depending on the specific requirements of the application, higher spatial resolution ECs can be used, at the cost of higher computational cost and higher power budget [66].

**Hybrid pipelines:** Hybrid of complementary event and image sensors can remove some of their shortcomings while enabling the benefits. There also exists compact hybrid sensor solutions [1] sharing the same lens, which can be employed, for instance, for motion de-blurring as shown recently [33]. Therefore, alternatively, for energy efficient higher precision and detailed alignment, the coarse face pose estimation can activate a more precise frame-based image acquisition and processing, after a first detection, implementing an approach similar to progressive initialization [43].

## 8. Conclusions and Future Work

We showed for the first time face pose alignment with an EC, validated on a challenging EC dataset featuring large head pose variations at different speeds and large differences in face appearance. We devised an evaluation methodology which also takes EC sensor related issues into account. We applied motion detection to drive a cascaded tree ensemble regression for alignment. The proposed coarse pose alignment is thought as a preliminary processing for pose-free EC-based facial processing applications. Specifically, the use of EC sensors coupled with the proposed face alignment, can favour the development of EC-based algorithms for applications that mostly rely on temporal information extraction, such as voice activity detection, speech recognition or dynamics-based identity recognition.

Since ECs are motion sensitive, we made speed-dependent assessments and performed speed-balanced quantitative characterisation, to suppress the speed bias on the test set for better generalization. Our work targeted extremely efficient coarse alignment exploiting the characteristics of EC. The algorithm implementation is therefore tuned to such data. A comparison with the state of the art would require the translation of the event stream into frames and vice versa. The results would depend on the method used to generate frames from events, making the comparison dependent not only on the algorithm used for regression. For an assessment of the proposed pose predictor we therefore compared to human performance, exploiting the ground truth data from human annotators. In parts of the dataset there is significant disagreement between human annotators, due to absence of static texture measurements with event sensors, in these points, the errors of the predictor are more pronounced than inter-human disagreements both in terms of accuracy and precision. When the human disagreement rate is about 1%, the average predictor failure rate is about 2.7%. The failure rate increases to 3.15% for intense motion clips while human disagreement rate slightly goes up to 1.1%. On the other hand, with moderate motion clips, failure and disagreement rates are 0.42% and 0.36%, respectively. In general, both predictor and human performance deteriorates in correspondence of the slowest and fastest movements.

We characterised the predictor performance under different levels of initial scale-space localization uncertainty, to simulate face detectors/trackers of different precision. With increasing uncertainty, the alignment errors significantly increase. In addition, we investigated the performance of different levels of predictor complexity. When the given face location is relatively closer to the true coordinates, i.e., at low localization uncertainty, a simpler predictor obtains performance close to the human-baseline, except for the fastest and the slowest speed levels. To improve robustness to increased localisation uncertainty, the predictor must rely on more complex models. Decreasing the spatial resolution to as low as 29×37 pixels degrades the performance of about 1%, showing that EC-based pose alignment can work at very low spatial resolution.

In addition to the alignment quality, we also evaluated the time-complexity of the predictor, which is extremely low thanks to the use of the most efficient alignment approach in the literature. Our analysis shows that the alignment operation is the computationally dominant part and the motion detector is extremely lightweight. Since the alignment is activated only when there is motion, our method also helps to keep power consumption at lower levels in the absence of significant motion and is extremely appealing for always-on embedded processing. The average alignment rate in our dataset, after combining intense motion and moderate motion clips, is 32.2 Hz, instead of 50 Hz for fixed video-rate processing. Consequently, average time-complexity performance changes from 5 MFLOPs to 21 MFLOPs, depending on the level of localization uncertainty conditions.

Using EC as a novel vision modality for face pose alignment opens up a new area of future study. We believe there is a big room for improvements. More informative or more invariant pixel-event features as well as different predictor models can be explored. To deal with high initial localization uncertainty, and allow for the use of imprecise, but efficient, EC-based face detectors, joint face motion localization and detection can be developed. To better handle the slowest and fastest motion levels, speed-dependent prediction can be studied. Another future work could focus on improvements to computation efficiency, especially taking advantage of the highly sparse nature of EC data. Including tracking could be highly beneficial, though with additional challenges. While detection-based alignment is still needed at initialization and is crucial to prevent drifting [45,48], tracking can reduce alignment complexity by relying on previous predictions, and accuracy can be improved by exploiting temporal information and spatio-temporal continuity. Finally, higher precision alignment may be achieved by using more landmarks, if ground-truth of sufficient precision can be acquired, by incorporating intensity sensors or perhaps by the help of intensity reconstruction [34].

## Figures and Tables

**Table 1:** Pose-annotated clip category contents. Intense category includes faster, longer range and more frequent movements with multiple repetitions within a clip compared to moderate category.

Category	Actions	Clips	Length (min)
Intense head motion	rotations (roll, pitch, yaw), eye-blinks	54	4.3
Moderate head motion	rotations (roll, pitch, yaw), talking lips, eye-blinks	54	5.9
Total		108	10.2

**Table 2:** Pose initialization uncertainty ranges.

Uncertainty	Low	Medium	High
$r_{trl}$ (translation)	0.25	0.5	1.0
$r_{scl}$ (scale)	0.1	0.2	0.4

**Table 3:** Comparison of average prediction errors under low localization uncertainty with inter-human annotation disagreement errors for all, intense and moderate head motion clips (E-rate: average pose-event rate in Hz., Fail %: alignment failure percentage and P. Err.: alignment precision error).

	Prediction			Human Annotation	
Category	E-Rate (Hz)	Fail %	P. Err.	Fail %	P. Err.
All	32.2	2.73	0.13	1.02	0.12
Intense motion	36.3	3.15	0.13	1.10	0.12
Moderate motion	29.1	0.42	0.11	0.36	0.11

**Table 4:** Speed level comparison of prediction and human annotation in terms of failure percentages under low localization uncertainty for all, intense and moderate head motion clips. Each column displays prediction (Pr.) and human annotation (Hu.) failures for a RMS speed range in pix/s.

Category	[0,1)	[1,75)	[75,150)	[150,225)	[225,300)	[300,375)	[375,450)	avg.
	*Pr. – Hu.*	*Pr. – Hu.*	*Pr. – Hu.*	*Pr. – Hu.*	*Pr. – Hu.*	*Pr. – Hu.*	*Pr. – Hu.*	*Pr. – Hu.*
All	2.1 – 1.0	2.6 – 1.0	1.2 – 0.8	1.3 – 0.4	1.1 – 0.7	1.5 – 1.8	8.8 – 1.6	2.7 – 1.0
Intense motion	4.1 – 1.7	4.5 – 1.3	1.7 – 0.8	1.4 – 0.4	1.1 – 0.7	1.5 – 1.8	8.8 – 1.6	3.2 – 1.1
Moderate motion	1.1 – 0.7	1.7 – 0.8	0.0 – 0.6	0.0 – 0.0	0.0 – 0.0	–	–	0.4 – 0.4

**Table 5:** Alignment failure percentages for varying down-scales at the three levels of localization uncertainties. In bold, the optimal performance for a given uncertainty level.

**Spatial down-sampling**	$2^{-1}$	$2^{-2}$	$2^{-3}$
**Event-cell size**	3×3	5×5	9×9
**Resolution**	119×151	59×75	29×37
Low Uncertainty	3.1	**2.7**	4.0
Medium Uncertainty	14.6	13.7	**12.6**
High Uncertainty	26.9	25.6	**24.0**

**Table 6:** Alignment failure percentages for varying tree depths at the three levels of localization uncertainties. In bold, the optimal value for each uncertainty level.

Tree Depth	2	4	6	8
Low Uncertainty	4.2	**2.7**	3.0	3.2
Medium Uncertainty	14.7	10.3	**8.5**	9.7
High Uncertainty	26.4	18.9	**16.9**	17.3

**Table 7:** Optimal settings and resulting failure percentages at the three levels of localization uncertainty ranges.

	Prediction			Human
Method	Low	Medium	High	Annotators
Spatial down-sampling	1/4	1/8	1/8	–
Tree depth	4	6	6	–
Failure %	2.7	8.5	16.9	1.0

## Abbreviations

The following abbreviations are used in this manuscript:

EC	Event camera
ERT	Extremely randomized trees
DNN	Deep neural network
GPA	Generalized Procrustes analysis
FNR	False negative rate
RMS	Root mean square
